# Sensory modality- and load-dependent changes across cortical working memory representations

**DOI:** 10.1162/IMAG.a.1115

**Published:** 2026-02-11

**Authors:** Vivien Chopurian, Simon Weber, Thomas B. Christophel

**Affiliations:** Department of Psychology, Humboldt-Universität zu Berlin, Berlin, Germany; Bernstein Center for Computational Neuroscience Berlin and Berlin Center for Advanced Neuroimaging, Charité Universitätsmedizin Berlin, Corporate Member of the Freie Universität Berlin, Humboldt-Universität zu Berlin, and Berlin Institute of Health, Berlin, Germany; Research Training Group “Extrospection” and Berlin School of Mind and Brain, Humboldt-Universität zu Berlin, Berlin, Germany

**Keywords:** fMRI, working memory, capacity, MVPA

## Abstract

While distributed cortical areas represent working memory contents, their necessity for memory maintenance has been questioned. Here, we examined the differential effects of maintaining multiple items on the neural information across cortical regions. In each trial of the fMRI experiment, participants (n = 81) had to memorize two items, each either an orientation or a pure pitch, for 13.8 s and continuously recalled the target after the delay. We kept the overall working memory load constant, but varied the sensory modality of each item to vary the effective visual load. We find significant information for orientations in visual, parietal, and frontal areas. We show that increasing visual load decreased behavioural recall performance and orientation-specific information in visual cortex. Parietal areas were affected only early in the delay, whilst frontal representations were unaffected by load. Simulations show that this drop in decodable information is best interpreted as a drop in mnemonic information represented by multivoxel patterns. Our results provide evidence for shared labour of visual cortices, where maintaining two versus one orientation leads to a loss in representational fidelity, and anterior cortices, where multiple items could be represented in a more robust but less precise format.

## 1 Introduction

Working memory capacity is limited. The precision of recall decreases as the number of items to remember increases (Gorgoraptis et al., 2011; Ma et al., 2014). In neuroimaging studies, the load-dependent decrease in recall performance is accompanied by a decline in the strength of visual working memory representations (Emrich et al., 2013; Sprague et al., 2014; Sutterer et al., 2019). While this evidence demonstrates a link between the ability to decode cortical representations and the capacity to memorize information, it remains unclear whether different cortical regions play distinct roles as memory load increases.

Previous studies highlight a distributed network of sensory, parietal, and frontal regions to maintain memorized contents during working memory delay (Christophel et al., 2017; D’Esposito, 2007; Fuster, 1998; Xu, 2023). For single visual items, studies have found correlations between decodable information in visual cortex and recall performance across participants and trials (Ester et al., 2013; Hallenbeck et al., 2021; Weber et al., 2024). The neural selectivity in visual cortex might enable the maintenance of sensory-like representations (Ester et al., 2013; Harrison & Tong, 2009; Serences et al., 2009). However, these representations could be modulated by incoming visual input, as they have been found to be susceptible to visual distractors, leading to reduced delay-period decoding if taxing distractors also affected working memory recall (Hallenbeck et al., 2021; Lorenc et al., 2018; Rademaker et al., 2019). Imaging and single-cell studies also demonstrated the maintenance of item-specific representations in parietal and prefrontal regions (Bettencourt & Xu, 2016; Ester et al., 2015; Funahashi et al., 1989; Goldman-Rakic, 1995) and in non-human primates, persistent delay activity in prefrontal cortex directly correlated with behavioural performance (Constantinidis et al., 2001; Wimmer et al., 2014). Presumably, anterior regions maintain abstracted, sensory-modality independent mnemonic representations (Leavitt et al., 2018; Spitzer & Blankenburg, 2012; Vergara et al., 2016), and appear to be protected from interference during visual distraction (Bettencourt & Xu, 2016; Lorenc et al., 2018; Rademaker et al., 2019). Furthermore, frontoparietal regions maintained information for multiple items independent of priority, whereas only the prioritized item was decodable from visual cortex (Christophel et al., 2018). Taken together, visual cortex and frontoparietal regions have been found to maintain item-specific mnemonic information, but the difference in distractor susceptibility across these regions could suggest different roles for the working memory storage of multiple items.

If multiple low-level visual items have to be encoded and maintained in working memory, their representations are likely to compete for resources in visual cortex (Franconeri et al., 2013). Prior work demonstrates that the extent of cortical overlap of multiple representations predicts the degree to which memory recall is impaired (Cohen et al., 2014). Therefore, we assume that working memory contents from different sensory modalities should interfere less because these representations share little overlap within sensory regions (Baddeley, 2003; Kiyonaga & D’Esposito, 2020). The recruitment of abstract anterior representations could reduce interference by helping to separate similar sensory representations (Overkott & Souza, 2023; Wing et al., 2022).

In this study, we investigated the effect of visual working memory load on these distributed cortical representations. We show, by keeping the overall working memory load constant at two items but varying the sensory modality of each item, that increasing visual working memory load selectively reduced continuous mnemonic information about items present in visual cortex (V1–V3), but less in intraparietal sulcus (IPS) and precentral sulcus (sPCS).

## 2 Methods

### 2.1 Participants

Eighty-six participants were scanned. Four datasets with incomplete recordings, resulting from participant dropouts, and one dataset with image artifacts were removed from the analysis. Our final sample consisted of 81 participants (48 female, 4 left handed, $mean_{age}$ = 25, $sd_{age}$ = 4). All participants provided their written informed consent and were compensated with 10€ per hour for their participation. The study was approved by the ethics committee of the Humboldt-Universität zu Berlin (2022-40) and conducted according to the principles of the Declaration of Helsinki (World Medical Association, 2013).

### 2.2 Task and procedure

**Fig. 1.**
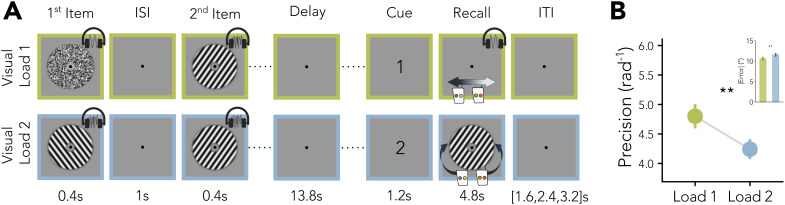
Task setup and behavioural results. (A) Example experimental trials with varying visual load. The top row shows a visual Load 1 trial, where items from both modalities are shown and the target modality is auditory. The bottom row shows a visual Load 2 trial, where both items are visual. Auditory Load 2 trials (not shown) are included in the design to balance the behavioural task. Visual samples were presented jointly with auditory noise and auditory samples were presented with visual masks. (B) Behavioural results for visual targets. Inset shows absolute error for the visual load conditions. Asterisks indicate significance levels of **p<0.01 . Error bars show the SEM.

In each trial of the delayed-estimation task, participants had to memorize two sequentially presented items (0.4 s, inter-stimulus interval: 1 s), either two visual orientation stimuli, two auditory pitch stimuli, or one of each. Crucially, this means that overall working memory load was constant at two items, but unisensory visual load varied between one or two items (see Fig. 1). We focused all our analyses on decoding visual stimuli but included the auditory–auditory condition (auditory Load 2) to balance the design behaviourally. Whenever a sample of a given modality was presented, the other modality was masked. After an extended delay (13.8 s), a visual retro-cue (“1” or “2” for 1.2 s) indicated whether the first or second item had to be recalled. During the continuous recall phase (4.8 s), participants had to adjust a probe orientation or pitch to match the target item by pressing the inner left and right button, and after confirming their response the fixation dot turned green. The next trial started after a brief period of fixation (1.6, 2.4, or 3.2 s).

The experiment consisted of 192 trials, divided in 4 runs, with short breaks in between. The item modality was counterbalanced across cued and non-cued items, 12 orientations and pitches were counterbalanced for each modality and randomized for the cued and non-cued items (see section 2.3). Thus, the two orientations (cued and non-cued) were independent of one another. We randomized the experimental conditions within each scanner run to minimize effects of biased training sets for the cross-validation procedure (see section 2.7). Before the main experiment, participants read instructions and completed 12 training trials during the anatomical scan. During these training trials, participants received feedback about their performance as the angular difference between their response and the target item.

### 2.3 Stimuli

Stimuli were generated with MATLAB and Psychtoolbox-3 (Brainard & Vision, 1997). The background color was set to grey (RGB [128,128,128]). The fixation dot (radius = 0.1°) was centred on the screen. Orientation stimuli and masks were presented as annuli with a total diameter of 7°, an inner diameter of 1°, and a Gaussian blur (Gaussian kernel = 1°, sd = 0.5°) on all edges. The visual stimuli consisted of 12 discrete orientations, from 7.5° to 172.5° with a 15° distance, excluding the cardinal axes. Participants were generally not aware of the exact number of orientation stimuli (as indicated by a post-experiment questionnaire). The spatial frequency was set to one cycle per degree. Visual masks were generated by filtering white noise to only include spatial frequencies between one and four cycles per degree (code from Rademaker et al. (2019)). During the recall, participants could select their response out of orientations between 1° to 180°, with a precision of 1°. For the auditory stimuli we used 12 pure tones with a distance of 1 semitone, between 251.63 Hz and 493.88 Hz, with a logarithmic ramp up and ramp down. White noise was chosen for the auditory masks. The volume was adjusted for participants’ comfort level. During the recall period, a random pitch was played, and participants could select a response between 246.93 Hz and 523.24 Hz, with a precision of $2^{\pm 1/70}$ . A longer button press increased the response step size, making it easier for participants to select their response in both modalities.

### 2.4 Magnetic resonance imaging

Imaging data were recorded on two 3-Tesla Siemens Prisma MRI scanners with a 64-channel head coil. The first 40 participants were scanned at the Berlin Center for Advanced Neuroscience (Charité Berlin, Germany) with the Syngo MR VE11C operating system, a viewing distance of 148 cm, and screen of dimensions of 52 x 39 cm. The subsequent 41 participants were scanned at the Center for Cognitive Neuroscience Berlin (Freie Universität Berlin, Germany) with the Syngo MR XA30 operating system, a viewing distance of 106 cm, and screen dimensions of 48 x 27.2 cm. Stimulus size was adjusted according to the screen size and viewing distance, so that all participants received the same visual input. The decoding results for both scanners did not differ qualitatively from the combined results. At the beginning of each session, we recorded T1-weighted MPRAGE structural image (208 sagittal slices, TR = 2400 ms, TE = 2.22 ms, flip angle = 8°, voxel size = 0.8 mm^3^, FOV = 240*256 mm). For each of the four runs, we recorded functional images with a multi-band accelerated echo planar imaging sequence, with a multi-band factor of 8 (72 sagittal slices, TR = 800 ms, TE = 37 ms, flip angle = 52°, voxel size = 2 mm^3^, 0 mm inter-slice gap, FOV = 208*208 mm).

### 2.5 Preprocessing

All preprocessing steps and analyses were performed in MATLAB, using SPM12 (Ashburner et al., 2014), The Decoding Toolbox Version 3.999E2 (Hebart et al., 2015), and in-house software. First, we converted the acquired DICOM images into NIfTI format. The intensity-normalized anatomical image (T1) was coregistered to the first functional image of the first run for each participant. During segmentation, we estimated the normalization parameters to the Montreal Neurological Institute (MNI) standard space. Functional images were spatially realigned to the first image of the first run and resliced. To remove slow signal drifts accumulating across each run, we applied a cubic spline interpolation to each voxel’s time series (n_nodes_ = 24). Afterwards, we applied temporal smoothing by running a moving average of five TRs. We used the detrending algorithm and moving average as described in Weber et al. (2024).

### 2.6 Identifying ROIs

We focused on the distribution of representations across three regions of interest (ROI) using probabilistic maps of retinotopic regions of the human neocortex (Wang et al., 2015). To generate a mask for visual cortex, we combined maps for areas V1 to V3. To obtain the bilateral IPS mask, we combined IPS0 to IPS5 from the same probabilistic atlas, and similarly, for the sPCS (FEF), left and right hemispheres were combined into one mask. We transformed these maps into single-participant space by applying the inverse normalization parameters estimated during preprocessing. We excluded voxels that had less than a 10% probability to belong to each ROI, respectively. Secondly, we obtained activity-driven maps by estimating participant-level univariate GLMs for onsets of every orientation presented. After contrasting visual (orientation) activation and baseline, we selected the 1000 most active voxels. We selected univariate activated voxels, as previous work suggests that multivariate item-specific effects may emerge because of voxel-wise item-selective univariate signals (Albers et al., 2018; Haynes, 2015). Decoding results without functional voxel selection reflect the main results (Supplementary Fig. S3).

### 2.7 fMRI analyses: pSVR

We analyzed fMRI data using support vector regression (SVR, Chang & Lin (2011)) to continuously reconstruct memorized orientations. The orientation labels were transformed from the 0°–180° space into radians from -pi to pi. The angular orientation labels (θ) were then projected into sine (y) and cosine labels (x), which provided a linear measure of the circular orientations. We then predicted these new labels (x,  y ) separately from the multivariate voxel pattern in each ROI with an SVR with a radial basis function kernel (periodic SVR, Weber et al. (2024)). In each cross-validation fold, we rescaled the train data voxel activation to 0–1 and applied the estimated scaling parameters to the test data. The orientation label was reconstructed by using the four-quadrant inverse tangent:

$$\hat{\theta }=atan2\left(\hat{y},\hat{x}\right).$$ (1)

We calculated the circular absolute difference between presented and predicted orientation label:

$$\Delta x=\left|{\left(\theta -\hat{\theta }\right)}_{circular}\right|$$ (2)

and this difference was then transformed into a feature continuous accuracy (FCA) above chance, in line with the existing literature applying pSVR (Degutis et al., 2024; Weber et al., 2024). Please note that when comparing the decoding measure with prior work, the absolute value of accuracy (or error, or correlation) is affected by the number of repetitions within participants, behavioural performance, and the overall task design.

$$FCA=100-\frac{\Delta x}{pi}\times 100.$$ (3)

We subtracted 50% from the resulting FCA, so that 0% refers to chance level decoding or an angular error of 45° (for stimuli in 180° space). This conversion means that values larger than chance level imply a smaller decoding error. Supplementary Figure S1 shows the trial- and time point-wise decoding error distribution of three example datasets with high to poor overall decoding performance.

**Fig. 2.**
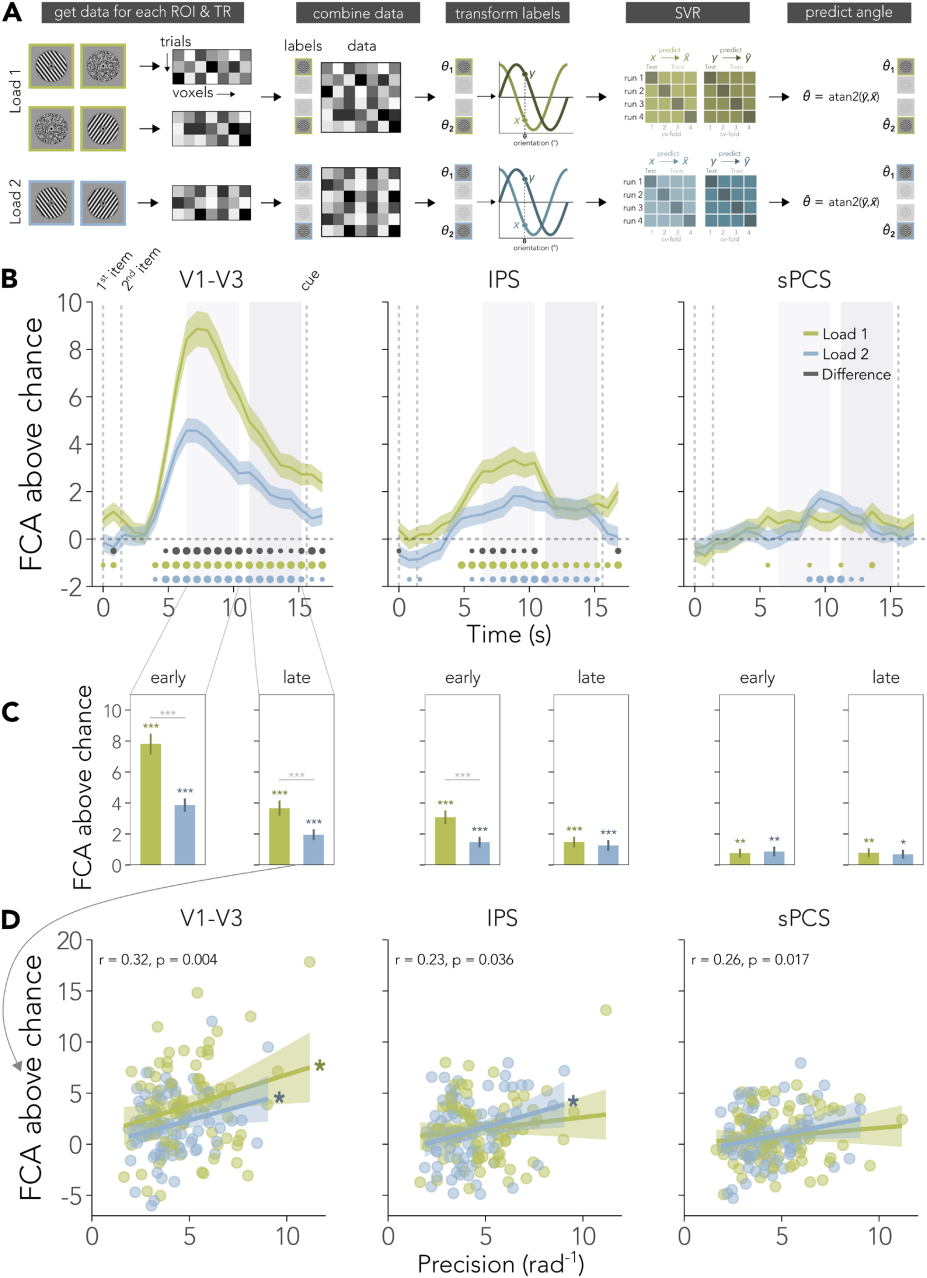
Decoding memorized orientations across regions. (A) Analysis steps for each region (ROI), time point (TR), and load condition. We converted the orientation labels into sine (y) and cosine (x) labels, which were predicted by individual support vector regression (SVR) models. The difference between the orientation labels and predicted labels was converted into feature continuous accuracy (FCA). A FCA of 0% refers to chance level decoding error of 45° (Weber et al., 2024). See section 2 for details. (B) Time course of item-specific decoding accuracy for each visual load condition in all three ROIs. Early (6.4 s–10.4 s), where signals can be attributed to perceptual and mnemonic signals, and late delay (11.2 s–15.2 s), where signal can be attributed to working memory maintenance, were chosen to avoid overlap. (C) Average difference between Load 1 and Load 2 in early and late delay. Asterisks above each bar indicate uncorrected above chance significance. (D) Correlations between late delay FCA and recall precision. Correlation coefficient in figures refers to correlation across both conditions, while asterisks indicate significant correlations in the respective load condition. Dots (small, medium, large) at the bottom of (B) and asterisks in (C, D) indicate significance levels of *p<0.05 , **p<0.01, or ***p<0.001 . In (B, C) shaded ribbons and error bars show SEM and 95% confidence interval in (D).

All pSVR analyses were run for each ROI, load condition, and TR individually. On each cross-validation iteration, we trained on the trials of three runs and tested on the left-out run. Because our analyses were cross-validated within each participant, we increased the computational power for each load condition and combined all orientations, cued and non-cued, within each of the load conditions (Fig. 2A). That means that for the Load 2 condition, data for each trial was used for training and testing, once with each item’s label (see also section 2.9 below). For the temporal generalization analysis (Fig. 4), we trained the pSVR on each time point and tested on each time point. This was done for each load condition separately as well as across load conditions, where we, for instance, trained on each time point from Load 1 and tested on each time point from Load 2.

### 2.8 Statistical analyses

We used R (R Core Team, 2020; RStudio Team, 2019) (Version 4.4.2) for the statistical analyses and figures. We analysed the behavioural data for the orientation recall by calculating precision $\left(\frac{1}{sd}\text{ in radians}\right)$ of the angular difference between target orientation and response orientation for each condition and participant (Gorgoraptis et al., 2011). Since the orientation space is circular, we used the circular SD. We compared the average precision of each visual load condition with paired two-sided t-tests.

To test for significant differences in decoding accuracy between the load conditions (visual Loads 1 and 2), we used paired two-sided t-tests. In particular, we tested decoding accuracy during a period early in the delay, where we assume to find perceptual activation mixed with mnemonic representations (due to the haemodynamic lag), and a period later in the delay, where we assume to find information about mnemonic representations without a substantial influence of perceptual representations. We averaged decoding accuracy per participant in each of these two sections of the delay period (early delay: 6.4 s–10.4 s; late delay: 11.2 s–15.2 s). Then we tested for the difference between the load conditions within each delay period with paired two-sided t-tests and within each condition for decoding accuracy above chance (50%) with two-sided t-tests (Fig. 2C). We focused all our other analyses on the late delay. Where applicable, for example, when comparing results across ROIs and load conditions, we corrected for multiple comparisons with Bonferroni correction. For descriptive purposes, we also compared decoding accuracy with chance level and between conditions (Fig. 2B) with two-sided t-tests for each time point (22 TRs), from the onset of the first stimulus to recall onset. The reported correlation coefficients (e.g., Fig. 2D) refer to Pearson’s correlation. Correlations were compared with the R package cocor (Diedenhofen & Musch, 2015), where we report the *p*-values for Pearson and Filon’s z, and the partial correlations were calculated with the R package ppcor (Kim, 2015).

### 2.9 Simulations

To determine how different analyses choices and decodable information content may influence the results, we simulated multivoxel data for load conditions 1 and 2 with varying levels of signal strength. We simulated data for a single time point with the same experimental parameters as in our fMRI experiment (n participants = 81, n runs = 4, n orientations = 12, n voxels = 1000) and ran each simulation 1000 times. First, we defined the similarity of the orientation space with 12 cosine functions raised to the power of 11 (Sprague et al., 2019), covering the whole orientation space (0°–180°) and their centers set at each stimulus label. Next, we defined a weight matrix (W) for each participant. This weight matrix models the voxel sensitivity for the orientation space and stayed constant for each participant throughout the iterations. The noise is sampled from a normal distribution with mean μ=0 and σ=1 (Welvaert & Rosseel, 2014):

$$noise∼N(\mu ,\sigma^{2}).$$ (4)

We simulated the different load conditions with different noiseFactors (e.g., 0.1, 5, 12.5) to a signalFactor of 1, which weights the noise and signal, respectively, to approximate our empirical level of decoding accuracy. The simulated multivariate voxel pattern for a single orientation can thus be described as:

activity=(W×basisfunctions)×signalFactor (5)

data=activity+noise×noiseFactor. (6)

**Fig. 3.**
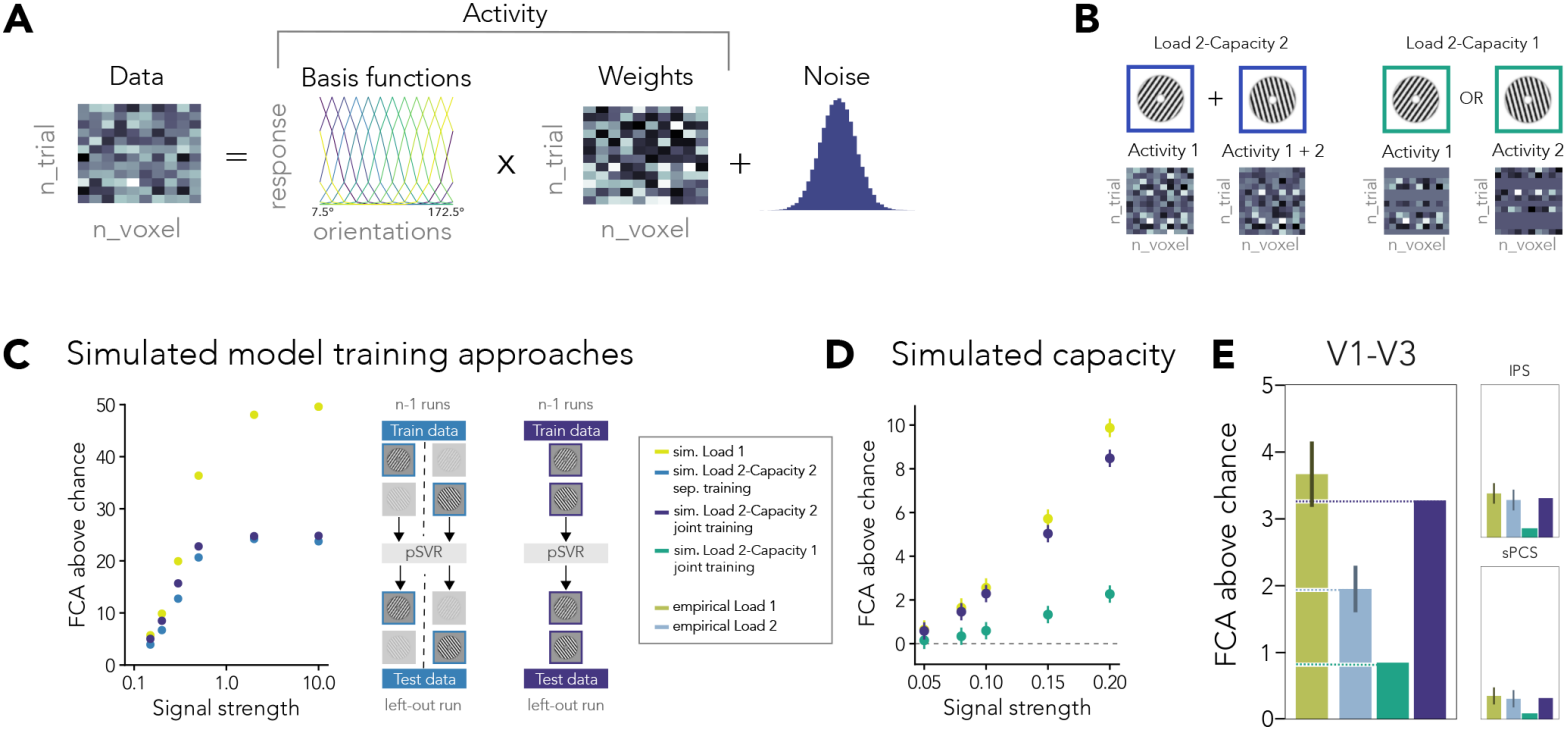
Simulations of model training approaches and capacity limits. (A) Schematic of multivariate data generation. (B) Multivariate patterns for Load 2 conditions. Addition of activity patterns for Load 2–Capacity 2 or random selection of a single pattern per trial for Load 2–Capacity 1. (C) Results for simulated Load 1 and Load 2 conditions with different model training approaches under varying signal strength (log scale). Load 2–separate training refers to the separate decoding of the two orientation labels presented in that condition. Load 2–joint refers to the decoding scheme used in our experiment, where we include both labels in one model. (D) Decoding results for simulated data, where either both items (Load 2–Capacity 2) or only one item (Load 2–Capacity 1) is maintained in the multivariate pattern. (E) Comparison of empirical decoding results (late delay) with simulated Load 2 conditions. These were matched to the empirical Load 1 based on the signal strength (see Table 1). Error bars show SEM.

For Figure 3C, we simulated a Load 1 condition across different signal strength levels. To evaluate how training and testing on both orientation labels in Load 2 impact the decoding accuracy, we compared our analysis strategy of decoding both items jointly to a model training approach where the first and second items are decoded separately.

**Table 1.** Simulated Load 1/Load 2 ratios compared with empirical data.^a^

Signal strength	sim. Load 2–Capacity 2	sim. Load 2–Capacity 1	Empirical data
0.1	1.12	4.32	V1-V3: 1.89
0.08	1.12	4.86	IPS: 1.18
0.05	1.10	4.24	sPCS: 1.14

a Signal strength refers to the ratio between the noise factors to a signal factor of 1.

Next, to determine whether the drop in visual cortex decoding accuracy is evidence of visual cortex only maintaining one item in the focus of attention (Garavan, 1998; McElree, 2006; Oberauer, 2002; Sutterer et al., 2019), we simulated two Load 2 conditions (Fig. 3B). First, we simulated the effect of additive patterns with increased working memory load (Load 2–Capacity 2), where we generated the multivoxel pattern for each orientation label and then added two patterns, assuming a simplified superpositional code for multiple items (Botvinick & Plaut, 2006; Sutterer et al., 2019):

$$\begin{array}{l} data_{Load2}=\left(activity_{orientation1}+activity_{orientation2}\right)\\ \;\;\;\;\;\;\;\;\;\;\;\;\;\;\;\;\;+noise\times noiseFactor. \end{array}$$ (7)

Additionally, we simulated a capacity of 1 by randomly selecting the pattern for one of the items in each trial before adding the two patterns (Load 2–Capacity 1; see Sutterer et al. (2019)).

In our data, the average decoding accuracy drops from Load 1 to Load 2 by a factor of ~2 in V1–V3. To explore which simulated Load 2 condition matches this ratio, we calculated the ratio of Load 1/Load 2 for each of our simulated load conditions and signal strengths. For Figure 3E, we divided the empirical decoding accuracy in Load 1 by this ratio. We selected different signal strength conditions for each ROI based on the approximate empirical Load 1 decoding accuracy.

### 2.10 Temporal generalization analysis

**Fig. 4.**
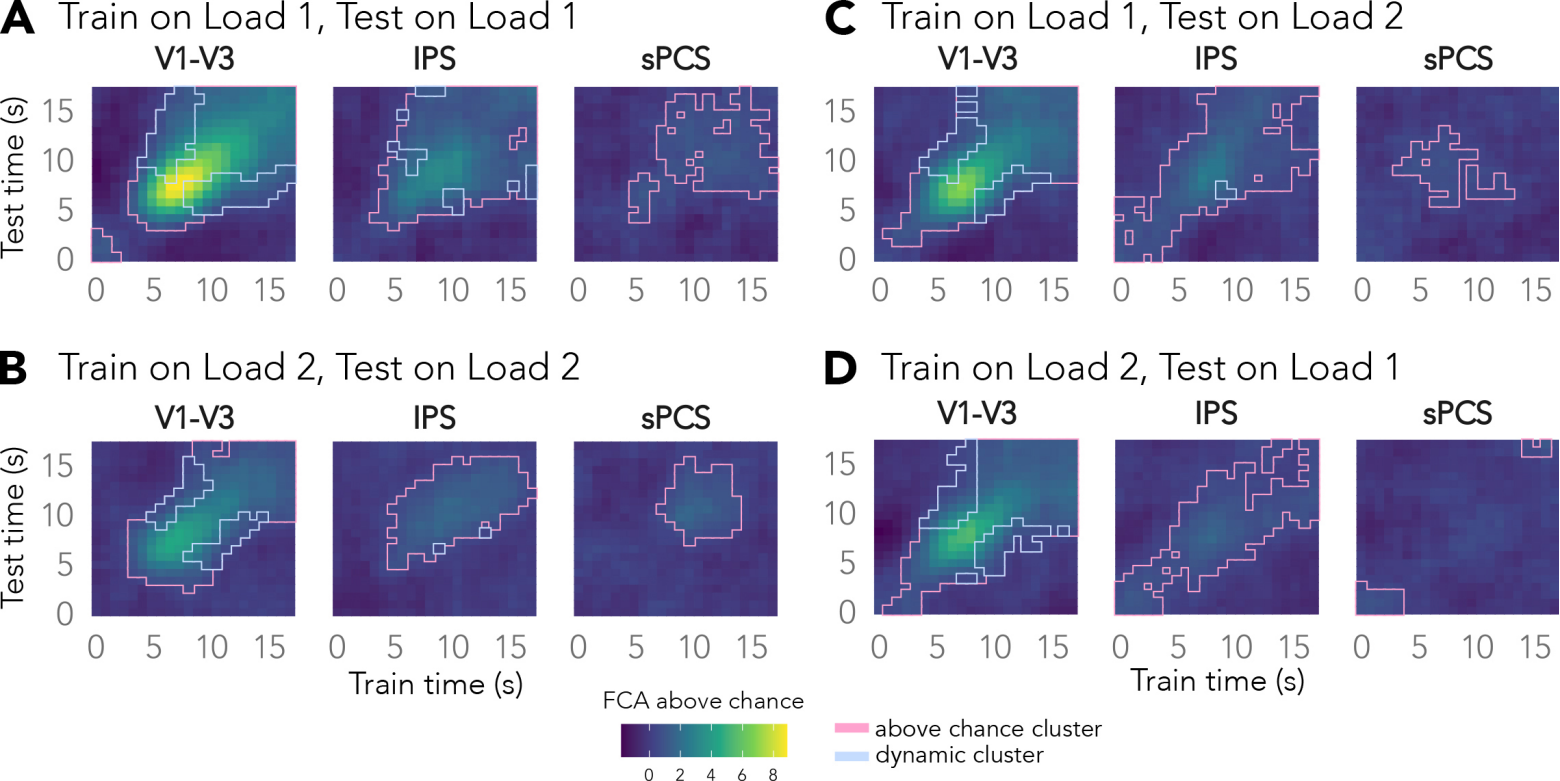
Mnemonic representations generalize between conditions in visual and parietal areas. Results for cross-condition temporal generalization, where we trained on each time point in each load condition and tested on each time point in the other load condition. Cross-temporal decoding for (A) Visual Load 1 and (B) Visual Load 2. Cross-temporal decoding when (C) training on TRs from Load 1 and testing on TRs from Load 2, and (D) training on TRs from Load 2 and testing on TRs from Load 1. 0 s indicate the first item onset.

To assess whether the neural code is generalizable across time and load conditions, we conducted a temporal cross-decoding and cross-condition analysis. Here, we trained on data on one time point and tested on data from all other time points, within and across the load conditions (Fig. 4). As in the main analysis, the pSVR was cross-validated by training on three runs and testing on the left-out run. We applied a cluster-based test (Maris & Oostenveld, 2007) to determine above-chance clusters, where the neural code was generalizable across time. First, we generated a null distribution by randomly assigning the sign (an FCA of 0 refers to chance level) of all elements of the decoding accuracy matrix. Then we calculated the summed t-value for the largest randomly occurring cluster and repeated this procedure 1000 times. The resulting null distribution cluster was then compared with our empirical summed t-value with a threshold of *p* < 0.05 to determine significance (Degutis et al., 2024).

Dynamic clusters were elements of the temporal cross-decoding matrix where the neural code at one time point did not generalize to another time point, within and across conditions. This was defined as off-diagonal elements with significantly lower decoding accuracy than their two corresponding diagonal elements ($a_{ij}<a_{ii}$ and $a_{ij}<a_{jj}$ ). To test whether the accuracy was significantly smaller, each of these two conditions was tested with a cluster-based permutation test. We subtracted the diagonal element from the off-diagonal element ($a_{ij}-a_{ii}$ and $a_{ij}-a_{jj}$ ) and applied the same sign permutation test, as mentioned above, for both comparisons. Only if both tests were significant (*p* < 0.05) and the element was part of the above-chance cluster, the element was defined as dynamic (Degutis et al., 2024; Li & Curtis, 2023; Spaak et al., 2017).

### 2.11 Bugs

For the first 11 participants, the number of images recorded in each run was set to 1448 instead of 1460, hence ending immediately after the last trial. Nineteen participants (pattern 1: 11, pattern 2: 8) had the same randomization for the trial order due to a glitch in the code. The results did not change qualitatively after removing these participants from the analyses.

## 3 Results

### 3.1 Visual load reduces mnemonic information in visual cortex

On trials with visual recall, participants performed better on trials where only the target item was an orientation and the other item was a pitch (visual Load 1, $precision=4.80rad^{-1}$ ) compared with the visual Load 2 condition (visual Load 2, $precision=4.24rad^{-1},\;t_{\left(80\right)}=3.36,\;p=0.001$ , Fig. 1B). To investigate this visual load effect on the distributed cortical representations, we decoded orientations continuously with a periodic support vector regression (pSVR Weber et al. (2024), see section 2 and Fig. 2A) from fMRI data. While all three regions of interest, the visual cortex (V1–V3), intraparietal sulcus (IPS), and precentral sulcus (sPCS), show information about orientations, the feature continuous accuracy (FCA) in each region changed with load and across time (Fig. 2B).

The decoding accuracy in visual cortex was affected by visual load throughout the trial. Our results showed main effects of load ($F_{\left(1,80\right)}=47.57,p<0.0001$ ) and time ($F_{\left(1,80\right)}=54.46,p<0.0001$ ), as well as interactions between load and time ($F_{\left(1,80\right)}=14.16,p<0.001$ ) on decoding accuracy. Early in the delay (6.4 s–10.4 s after first item onset), decoding accuracy decreased with increased visual working memory load ($t_{\left(80\right)}=7.53,p<0.0001$ , Bonferroni corrected). In this early delay phase, cortical representations can be assumed to be a mixture of perceptual and mnemonic representations due to the haemodynamic lag. However, the same pattern of results is also present late in the delay (11.2–15.2 s after first item onset), when signals could be attributed to working memory signals ($t_{\left(80\right)}=3.52,p=0.002$ , Bonferroni corrected). While in both conditions decoding accuracy dropped from early to late delay (Load 1: $t_{\left(80\right)}=7.19,p<0.0001$ , Load 2: $t_{\left(80\right)}=4.52,p<0.0001$ , Bonferroni corrected), it never dropped to chance level (Fig. 2B). On average, we found above chance decoding in both conditions in the early delay (Load 1: $t_{\left(80\right)}=11.71,p<0.0001$ , Load 2: $t_{\left(80\right)}=9.00,p<0.0001$ , uncorrected) and late delay (Load 1: $t_{\left(80\right)}=7.50,p<0.0001$ , Load 2: $t_{\left(80\right)}=5.61,p<0.0001$ , uncorrected). Supplementary Figure S2 shows that the difference in visual cortex decoding accuracy between Load 1 and Load 2 is robust to different model training approaches. The difference remains when the pSVR is trained separately on cued and non-cued orientations within each load condition, as well as when trained and tested on all orientations from both load conditions combined.

Frontoparietal regions were not affected by load late in the delay. In IPS, our analysis revealed main effects of load ($F_{\left(1,80\right)}=7.07,p=0.009$ ) and time ($F_{\left(1,80\right)}=7.81,p=0.006$ ), as well as an interaction between load and time ($F_{\left(1,80\right)}=5.39,p=0.023$ ) on decoding accuracy. Here, decoding accuracy was reduced in visual Load 2 compared with Load 1 early, ($t_{\left(80\right)}=-3.50,p=0.002$ , Bonferroni corrected), but not late in the delay (p=1.0 , Bonferroni corrected). There was a significant difference in the change in decoding accuracy across time between load conditions. While the decoding accuracy for representations in the visual Load 2 condition showed no difference between the early and late delay period in IPS (p=1 ), the decoding accuracy for the single visual item dropped from early to late delay ($t_{\left(80\right)}=-3.29,p=0.004$ , Bonferroni corrected). There was above chance decoding in both conditions in the early delay (Load 1: $t_{\left(80\right)}=7.03,p<0.0001$ , Load 2: $t_{\left(80\right)}=4.29,p<0.0001$ , uncorrected) and late delay (Load 1: $t_{\left(80\right)}=4.32,p<0.0001$ , Load 2: $t_{\left(80\right)}=3.62,p=0.0005$ , uncorrected).

In sPCS, there was above chance decoding in both delay periods and conditions, but no significant main effects or interaction effects of load conditions and delay period (all p>0.7 ). There was no difference in decoding accuracy between load conditions in either early (p>0.8 , Bonferroni corrected) or late delay (p>0.8 , Bonferroni corrected). There was no difference between early and late delay for either visual Load 1 (p>0.9 ) or Load 2 (p>0.6 , Bonferroni corrected). Smaller differences between the load conditions might be detectable at subthreshold levels in IPS and sPCS in future work. Differences in IPS during the early phase of the trial could be either indicative of such a smaller load effect in memory, or perceptual interference between the items that has no lasting effect on memory representations.

We are interested in the relative difference between the load conditions within each ROI and do not directly compare the decoding accuracy across ROIs, as we assume different baseline levels due to differences in cortical architecture across regions (Bhandari et al., 2018; Haynes, 2015). However, rmANOVA interaction effects (Supplementary Table S1) indicate significant differences across ROIs for the load conditions and delay periods.

### 3.2 Decoding accuracy correlates with behavioural recall precision

Our results so far indicate that posterior as well as anterior regions store item-specific working memory representations. To assess the contributions of the three regions to the behavioural performance, we correlated late delay decoding accuracy with behavioural precision across participants. First, we used the overall decoding accuracy and overall behavioural precision values by averaging over both load conditions. Overall decoding accuracy correlated positively with overall behavioural precision in V1–V3 (r=0.32,p=0.004 ), IPS (r=0.23,p=0.036 ) and sPCS (r=0.26,p=0.017 , Fig. 2D). Next, we correlated the decoding and behavioural performance measures for each load conditions separately. Decodable information of orientations in visual cortex during the late delay correlated with behavioural performance separately in both visual load conditions ($r_{Load1}=0.25,\;p=0.023,\;r_{Load2}=0.25,\;p=0.022$ ). However, behavioural precision correlated significantly with decoding accuracy in IPS only in the Load 2 condition (r=0.27,p=0.016 ), but not in the Load 1 condition (r=0.13,p=0.24 ). In sPCS, decoding accuracy correlated overall with behavioural precision, but not significantly for either Load 1 (r=0.11,p=0.318 ) or Load 2 (r=0.21,p=0.055 ).

Averaged across both load conditions, decoding and behavioural performance were correlated in all three regions. Further analysis comparing these correlation coefficients across regions shows that they are not significantly different from one another (all comparisons p>0.4 ). It is possible that the decoding accuracy in these regions influences each other, as information may flow between them. A partial correlation analysis shows that the overall decoding accuracy in visual cortex correlates with overall behavioural precision when controlling for influence of information in IPS and sPCS together (r=0.25,p=0.025 ) and individually (controlled for IPS: r=0.24,p=0.03 , sPCS: r=0.29,p=0.008 ). However, the correlation in IPS with behavioural precision was no longer significant when controlling for the information content in V1–V3 and sPCS (all p>0.1 ). When controlling for the contribution of V1–V3, the correlation between behavioural precision and decoding accuracy in sPCS was still significant (r=0.23,p=0.036 ), but not when controlling for IPS (p>0.07 ) or both (p>0.06 ). Supplementary Figure S4 shows that the decoding accuracy is correlated between the three ROIs, and possibly suggests that the inter-regional correlations may vary with visual load.

### 3.3 Simulations indicate different information content in visual versus anterior regions

Our results indicate that in visual cortex, but less in frontoparietal areas, decoding accuracy for orientations is reduced when multiple items are maintained in working memory. But is our pSVR analysis able to differentiate overlapping representations and quantify the strength of the underlying representations? We simulated multivariate patterns with our experimental parameters and compared a Load 1 condition with different variants of a Load 2 condition (see section 2 and Fig. 3A and B).

To determine how our model training approach impacts the results under varying signal strengths, we simulated a Load 1 condition, where only one item is held in mind and two Load 2 conditions. In the “Load 2, separate training” condition, the two items were decoded separately and in “Load 2, joint training”, the items were decoded jointly, as in our main analysis. In Figure 3C, we show that with increasing signal strength, the decoding accuracy increases. However, in both model training approaches, the maximal decoding accuracy is around 25% above chance, while items in Load 1 can be hypothetically decoded with an FCA above chance of 50% (i.e., 100% accuracy). This shows that if both items are maintained in overlapping patterns, even for data where the signal is weighted 10 times more than the noise, the data itself limit the hypothetical maximum decoding accuracy. When the signal decreases, under conditions similar to our experimental data, we show that this drastic difference between load conditions does not carry over. Instead, the model training approaches achieve similar decoding accuracies for both load conditions. The effect of overlapping representations thus cannot account for the difference observed in our experimental data. For the Load 2 simulations, the joint training approach increases sensitivity slightly compared with the separate training approach. These results indicate that under more realistic signal strength levels, the reconstructed orientation likely represents an aggregate of both orientations. In Supplementary Figure S2A, we also show that the difference between load conditions persists selectively in visual cortex when applying the separate model training to our empirical data.

In these scenarios, we assume that in the Load 2 condition, the multivariate signal represents both items, reflecting a capacity of two (Load 2–Capacity 2). However, it might be possible that visual cortex only maintains one of the two items (Garavan, 1998; McElree, 2006). This effect may arise due to a similar mechanism previously implicated in prioritization of working memory items: When two items were maintained, only the one relevant for the upcoming task was easily decodable from visual cortex (Christophel et al., 2018). Both items, the one immediately relevant and the item relevant for a future task, were maintained in frontoparietal areas. To test this possibility, we simulated a Load 2 condition where on each trial one of the two orientations was randomly selected to be maintained, thus simulating a capacity of one (Load 1–Capacity 1; see also Sutterer et al. (2019)). While we varied the signal strength overall, we kept the strength of concurrent items the same in all simulations.

Our results for the comparison between Load 1 and Load 2–Capacity 2, where the two patterns overlap, show that there is only a minor drop in decoding accuracy. This indicates that we can indeed decode orientation information from overlapping patterns, if this small drop, due to a superpositional code, is accounted for. However, FCA dropped substantially when we simulated a capacity of 1 (Load 2–Capacity 1, Fig. 3C), indicating that a loss of information in the Load 2 condition can be identified with our methods under signal strength conditions similar to our experimental data.

How do these simulations compare with our empirical findings? We matched the simulated signal strength to the empirical Load 1 condition in each ROI and compared the ratio of FCA drop from Load 1 to Load 2 of our simulated results with our empirical results (Fig. 3E and section 2). In IPS and sPCS, our empirical results show high similarity to simulated results of a capacity of 2, where both items are concurrently maintained in working memory. This could suggest that there is little cost to storing additional visual items in these regions. However, neither of the simulated results matched our empirical results in visual cortex. Here, empirical FCA dropped below the level expected when two orientations would be maintained (Load 2–Capacity 2), but was higher than the FCA expected when only one of the items would be maintained (Load 2–Capacity 1). This suggests that more than one visual item can be maintained in V1–V3, but at a cost for the individual representations.

### 3.4 Shared representations in visual cortex, distinct mnemonic code in sPCS

Considering that we observed changes in decoding accuracy across delay phases, we tested whether the neural code changes across time. First, we probed whether the multi-voxel pattern at one time point generalizes to other time points, within each load condition. Importantly, cross-decoding scales with the overall decoding accuracy, which limits the interpretability of these results.

When a single orientation (Load 1) is maintained in memory, the neural codes seemed to generalize between early and late delay time points in all regions (Fig. 4A). In the Load 2 condition, temporal generalization is reduced, in accordance with the lower decoding accuracy early in V1–V3 and IPS (Fig. 4B). Temporal generalization in sPCS was limited to neighbouring time points later in the delay, potentially due to limitations in power.

In visual cortex, we find evidence for dynamic clusters, where off-diagonal accuracy is lower than both diagonal time points, in both load conditions (Fig. 4A, B). The neural code in visual cortex seemed to be changing dynamically from perception to maintenance period. The IPS showed fewer dynamic clusters, while no dynamic clusters were found in sPCS. As we cross-validated the decoder across runs (as in Fig. 2A) for all elements, the train and test data were always separated in time based on their actual acquisition time, so that systematic correlations across time do not explain these dynamic clusters (see Li & Curtis (2023)). However, while the dynamic clusters may reflect changes in the underlying neural code, they could be affected by a decreased signal-to-noise ratio over the course of the trial.

Next, we tested whether the neural code for orientations is shared between load conditions. We trained the pSVR on each time point in one load condition and tested on each time point in the other load condition. In visual and parietal cortex, we found significant cross-condition decoding (diagonal in Fig. 4C, D), suggesting that these representations generalized well across conditions. In sPCS, cross-condition decoding appears to be lower than within-condition decoding.

Overall, these results suggest that cortical working memory representations generalize across time, but not without loss of representational precision. It is further possible that the low cross-condition decoding in sPCS indicates different neural codes used for mnemonic representations in the two load conditions in this regions.

## 4 Discussion

Visual working memory representations are distributed across the cortex (Christophel et al., 2017). We investigated the effect of working memory load on these distributed representations by keeping the overall load constant at two items, but varying the sensory modality of each item. Our results suggest that the precision of visual working memory recall is limited by the resources available in visual cortex for the maintenance of multiple similar items. In contrast, increasing visual working memory load reduced item-specific information in IPS only during the early delay period and did not affect representations in sPCS. When averaged across both load conditions, the item-specific decoding accuracy in the late delay period correlated with recall precision in all three regions. In visual cortex, our cross-decoding analyses hint at dynamic representational changes from early to late time points, and potentially a shared neural code across load conditions, whereas frontal regions may represent orientations in distinct neural codes depending on the load condition.

In previous studies, increasing working memory load reduced the ability to decode neural representations of spatial locations or direction of motion (Emrich et al., 2013; Sprague et al., 2014; Sutterer et al., 2019). However, not all of these results can be selectively attributed to any region, as some findings were based on regions including voxels from early visual to prefrontal areas (Emrich et al., 2013) or posterior electrodes, including occipital and parietal sources (Sutterer et al., 2019). Region-specific changes were observed for spatial locations, where the strength of the representation decreased in visual and posterior parietal areas (Sprague et al., 2014). This indicates that different brain areas could contribute differently to working memory performance as load increases. Here, we dissociated visual, parietal, and frontal regions for the maintenance of orientation stimuli, which allows for the characterization of region-specific changes under varying load.

These previous studies, as well as our task, encouraged the precise recall of low-level visual features and thus precise neural encoding in visual areas. In our data, representations in visual cortex appear to be more strongly affected by interference between orientations, resulting in reduced decoding accuracy and decreased behavioural precision. Simulations of multivariate patterns for one and two orientations (see also Sutterer et al. (2019)) suggest that while both orientations (Load 2) can be maintained in working memory signals in visual cortex, it comes with a loss of representational strength. These results suggest that capacity limitations for detailed visual working memory may arise in visual cortex.

Frontoparietal representations were less affected by increased visual working memory load. This is in line with prior studies suggesting that parietal representations might support working memory maintenance in the face of increasing cognitive demand. For instance, while representations in the visual cortex can be disrupted by taxing perceptual distractors, those in IPS may be less affected (Bettencourt & Xu, 2016; Rademaker et al., 2019). When participants maintained two items, but prioritized only one at a time, both items were decodable from IPS and sPCS, whereas only the prioritized item was reliably decodable from the visual cortex (Christophel et al., 2018). However, for spatial locations, which likely rely more on parietal regions (Jerde et al., 2012), parietal representations were disrupted if a distractor was behaviourally relevant (Hallenbeck et al., 2021) or when multiple locations were maintained (Sprague et al., 2014). This suggests a flexible recruitment of different brain regions depending on the stimulus content and varying demands.

Although the unisensory load varied, our task required the maintenance of two items in each trial. This could selectively affect the storage in sensory areas, but less in frontoparietal regions, which have been implicated in maintaining modality independent mnemonic representations (Spitzer & Blankenburg, 2012). While this could suggest that the neural resource in these regions is similarly distributed across the visual and auditory items (visual Load 1) as it is across both visual items (visual Load 2), there seems to be little overlap between parietal regions for pitch and orientation information (Czoschke et al., 2021).

However, frontoparietal areas could store items in a more abstract format. Representing items in an abstracted format (Chunharas et al., 2023; Kwak & Curtis, 2022) would enable the maintenance of multiple items in distinct neural patterns, separating the items in stimulus and cortical space, which would reduce interference in frontoparietal areas. Our results show, in line with this idea, fewer dynamic clusters and lower cross-decoding between load conditions in these frontoparietal regions. A previous study suggested that visual areas appear to show stronger dynamic change in representations compared with frontoparietal areas when a single spatial location is maintained in working memory (Li & Curtis, 2023). Less representational changes suggest that the orientations may be stored in a more robust format in frontoparietal areas, even when load is increased.

Importantly, our temporal generalization and cross-decoding results are confounded by the strength of decoding accuracy in each region. While decoding accuracy is not comparable across ROIs, as two-way decoding of working memory contents showed a lower base rate in frontal areas than visual cortex (Bhandari et al., 2018), it is possible that decoders can pick up on the cortical retinotopy in visual cortex (Freeman et al., 2011), but possibly not the abstract features stored by frontal regions (Sreenivasan et al., 2014). If orientations are stored in a more abstract format in frontoparietal areas, and even visual areas during the late delay (Chunharas et al., 2023; Duan & Curtis, 2024; Kwak & Curtis, 2022; Yan et al., 2023), future studies could disentangle the effect of different neural codes on the maintenance of multiple items by applying encoding models.

In summary, we provide evidence for region-specific effects of visual working memory load and extend previous work to the often used orientation stimuli. Our results highlight the dissociable roles of visual, parietal, and frontal regions for working memory storage of multiple items.

## Supplementary Material

Supplementary Material

## Data Availability

The analysis code can be found here: https://osf.io/m5xv3. The data are not publicly available due to privacy or ethical restrictions. The data supporting this study are available upon reasonable request. Access to the de-identified data requires identification of a principal investigator and a brief written confirmation that no attempts will be made to re-identify participants and that their data privacy will be protected.
