# Supplementary material for: Sensory modality- and load-dependent changes across cortical working memory representations
Source: Imaging Neurosci (Camb). 2026 Feb 11;4:IMAG.a.1115. doi: 10.1162/IMAG.a.1115 (PMC12895694; doi:10.1162/IMAG.a.1115)
Supplement: Supplementary Material [file IMAG.a.1115_supp.pdf]

Supplementary Material

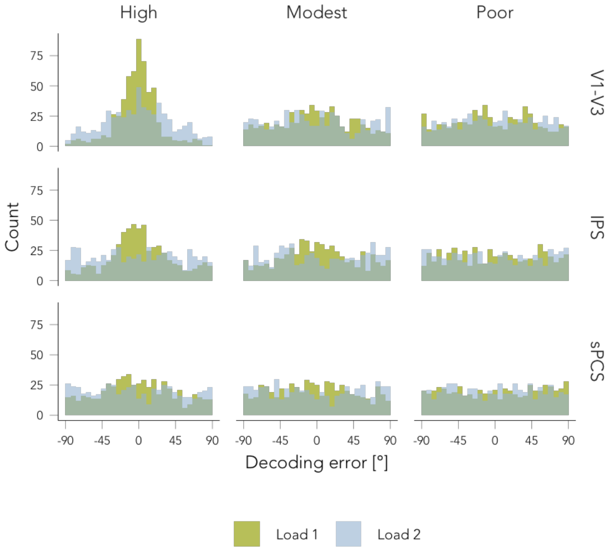

Supplementary Figure 1. Decoding error distributions for the early delay period for three example datasets with an overall *high*, *modest* or *poor* decoding accuracy. The participant-wise decoding performance was grouped into tertiles based on the overall decoding accuracy (FCA) across conditions and ROIs. *Poor* decoding performance refers to an FCA below the first tertile, *modest* to an FCA between the first and second tertile and *high* to an FCA above the second tertile. After selecting three example datasets, the decoding error in each time point of the early delay and each trial is plotted in degrees for the 180° orientation space.

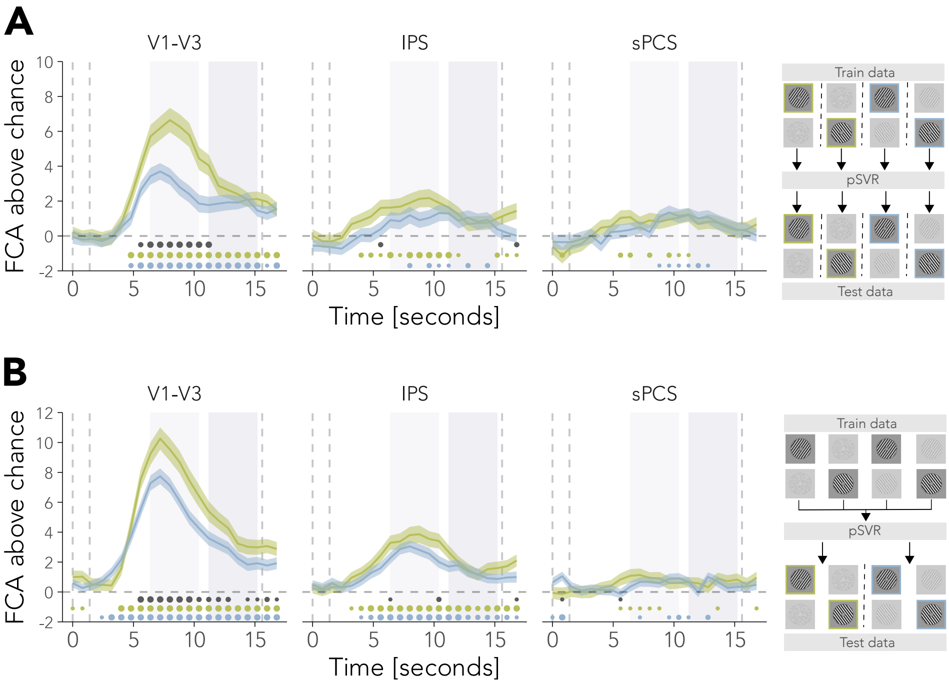

Supplementary Figure 2. Time courses for alternative decoding schemes. **A** shows results for training and testing on cued and non-cued orientations in each Load condition separately, then averaging the output for each Load condition after decoding. **B** shows results for training on all orientations, across both Load conditions, and then testing on the data from the Load conditions.

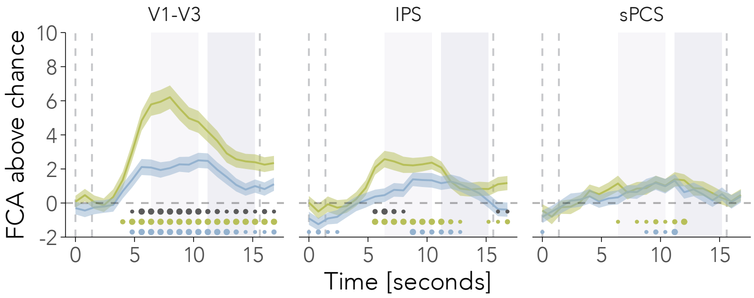

Supplementary Figure 3. Time course for decoding with all anatomical voxels.

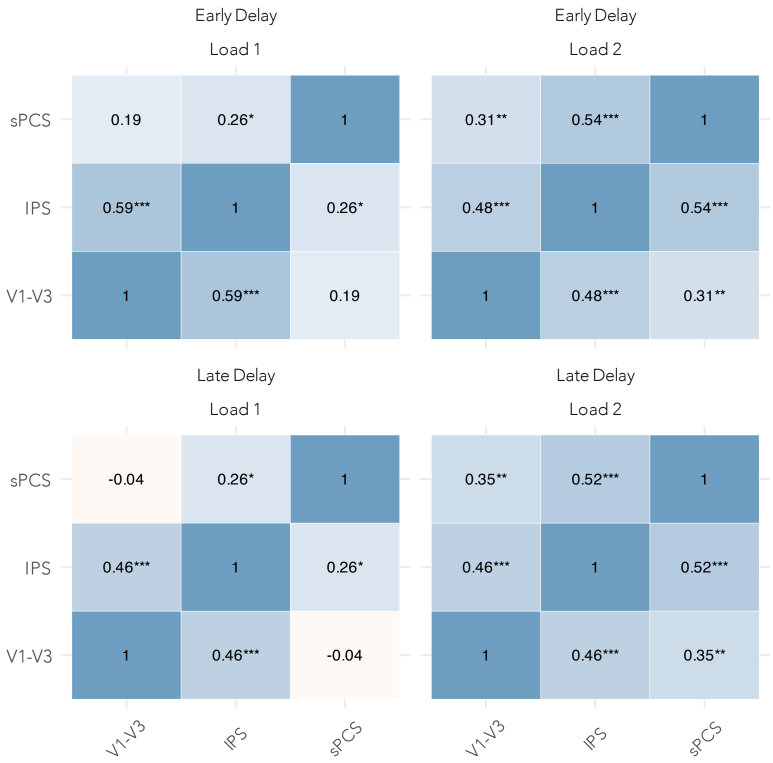

Supplementary Figure 4. Pearson correlation matrix between FCA in all three ROIs, in early and late delay and for each Load condition. Asterisks indicate significance levels of * $p < 0.05$, ** $p < 0.01$, *** $p < 0.001$.

	df	*F*	*P*
**ROI**	1.58, 126.03	81.334	< 0.001
**Load**	1.00, 80.00	22.481	< 0.001
**Delay**	1.00, 80.00	36.174	< 0.001
**ROI × Load**	1.65, 132.08	22.015	< 0.001
**ROI × Delay**	1.79, 143.51	24.004	< 0.001
**Load × Delay**	1.00, 80.00	7.389	0.008
**ROI × Load × Delay**	2.00,1 60.00	6.958	0.001

Supplementary Table 1. rmANOVA with ROI, load and delay as within-subject factors and decoding accuracy (FCA) as outcome.
